# Examining the Correlational Interaction of Environmental Fluoride and Selenium and Its Impact on Dental Fluorosis in Coal-Burning Regions of Southwest China

**DOI:** 10.3390/toxics13110940

**Published:** 2025-10-31

**Authors:** Na Yang, Jianying Wang, Longbo Li

**Affiliations:** 1 School of Public Health, Key Laboratory of Environmental Pollution Monitoring and Disease Control, Ministry of Education, Guizhou Medical University, Guiyang 561113, China; 2 Guizhou Geological Environment Monitoring Institute, Guiyang 550081, China

**Keywords:** fluoride, selenium, geochemical anomaly, Dean’s dental fluorosis index, spatial distribution pattern

## Abstract

Epidemiological and geochemical evidence suggests that coal-burning fluorosis in Southwest China is mechanistically linked to the presence of fluoride-rich geochemical anomalies. However, the severity of dental fluorosis does not consistently align with the distribution pattern of fluoride geochemistry, suggesting that other factors may interfere with the dose–effect relationship of fluorosis. To investigate the potential biotoxicity impacts of fluoride, this study conducted an analysis of soil fluoride–selenium spatial correlation in the central areas of coal-burning fluorosis in China. The results revealed that 59.1% of soil fluoride contents were more than the average soil fluoride content of China (800 mg·kg^−1^) and 77.9% of soil selenium contents were above 0.45 mg·kg^−1^. Soil fluoride (1.11 × 10^3^ mg·kg^−1^) and selenium contents (0.78 mg·kg^−1^) were significantly high states, but agricultural products and drinking water sources showed relatively low levels, not significantly influenced by soil conditions. The severity of fluorosis was evaluated using Dean’s dental fluorosis index (DFI). The spatial association of soil selenium or fluoride with DFI suggested that there was a reverse relationship between soil selenium or selenium/fluoride and the DFI. The generalized additive model (GAM) showed the onset of DFI correlated with soil fluoride content, showcasing a distinctive “W” pattern, while DFI decreased steeply or gradually as soil selenium content or selenium/fluoride ratio increased. In conclusion, our findings suggest that the geochemical anomaly of soil fluoride likely contributes to the occurrence of fluorosis. However, the significantly elevated levels of soil selenium might alleviate the severity of dental fluorosis to some extent.

## 1. Introduction

The issue of dental fluorosis in Southwest China was first reported by Lyth, who documented dental defects characterized by chalky bands or plaques and attributed these to excessive fluoride in drinking water [1]. Despite these early observations, significant scholarly and governmental attention did not emerge until the 1980s, leading to extensive epidemiological investigations and research [2]. These investigations revealed that the main cause of the widespread fluorosis was the consumption of contaminated fluoride-rich food. This unique phenomenon, particularly notable in Guizhou Province, China, has been termed “endemic coal-burning fluorosis”.

Recent studies have identified super-enriched fluoride in soil as a primary source of the dental fluorosis epidemic [3,4,5]. Geochemical anomalies suggested potential interactions with other compounds, which necessitated further investigation. Selenium, in particular, has been identified as significantly enriched in some endemic fluorosis areas [6]. Laboratory simulation experiments have demonstrated that selenium can inhibit fluoride toxicity [7,8,9]. Selenium plays a crucial role in the body’s defense against oxidative stress caused by fluoride exposure. Selenomethionine (SeMet) could alleviate fluoride-triggered inflammation and apoptosis in mice liver via blocking Parkin-mediated mitophagy [10]. More than 78% of fluoride and 97% of selenium in coal will be volatilized at 800 °C during coal firing [11]. Fluorosis can induce oxidative stress by leading to reactive oxygen species (ROS) generation. Selenium can eliminate ROS in direct and indirect manners [12]. However, evidence regarding the real-life impact of selenium on fluoride toxicity remains limited.

This study aims to analyze the spatial distribution patterns of fluoride and selenium in the central regions of coal-burning fluorosis areas in Southwest China. By establishing models corresponding to the severity of dental fluorosis, we explored the correlational relationship between fluoride and selenium in the environment. Understanding the spatial correlation of soil fluoride and selenium in these areas has significant implications for unraveling the etiology of dental fluorosis and developing effective mitigation strategies for affected populations. Through this research, we aim to contribute valuable insights that will inform public health interventions and advance our knowledge of the complex interplay between environmental factors and dental health in coal-burning fluorosis regions.

## 2. Materials and Methods

### 2.1. Study Area

**Figure 1:**
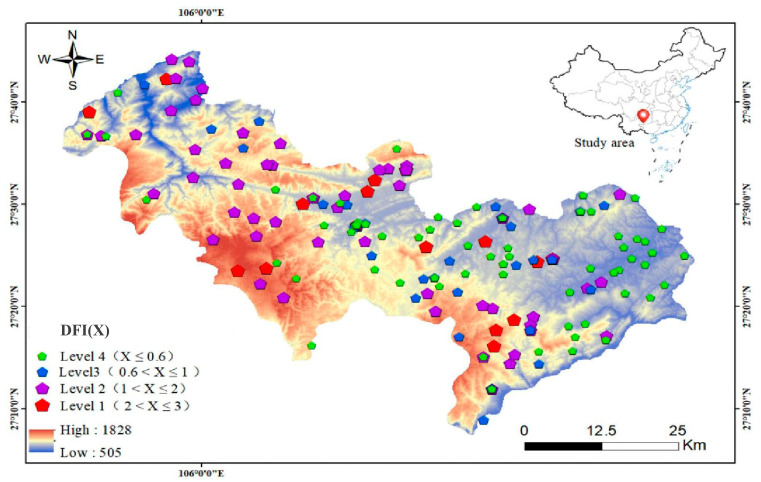
Spatial distribution of the severity of dental fluorosis in the coal-burning fluorosis areas.

The study area is located in a typical karst region of Guizhou Province, which is in the center of the coal-burning fluorosis area in China. It covers an area of 2528 km^2^ and includes 25 townships (towns) and 242 villages (communities). The arable land is distributed across the entire region. Figure 1 shows the severity of dental fluorosis. The data for the epidemic survey of dental fluorosis was obtained from the Guizhou Center for Disease Control and Prevention. Dental fluorosis in students aged 8–12 years was examined by trained medical staff using Dean’s method. The level of dental fluorosis was classified as questionable, very mild, mild, moderate, or severe based on the severity of enamel mineralization. Dean’s dental fluorosis index (DFI) is considered the gold standard of epidemiological research, and the relationship between DFI and the prevalence intensity is <0.4, 0.4–0.6, 0.6–1.0, 1.0–2.0, 2.0–3.0, and >3.0. Since the number with DFI of < 0.4, 0.4–0.6, and >3.0 is very small, DFI was divided into <0.6, 0.6–1.0, 1.0–2.0, and 2.0–3.0 in Figure 1. DFI was calculated as the following equation: DFI = (questionable × 0.5 + very mild × 1 + mild × 2 + moderate × 3 + severe × 4)/number of detected.

### 2.2. Sample Collection and Analysis

**Figure 2:**
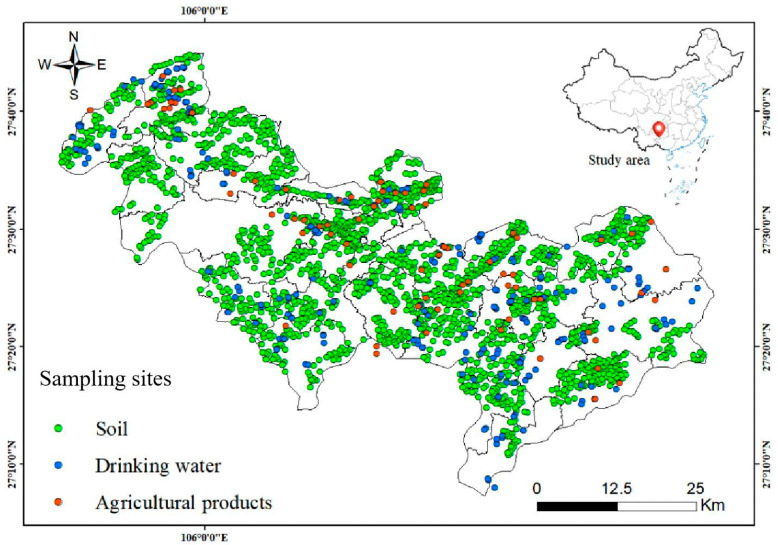
Spatial distribution of sampling sites including soil samples, drinking water samples, and agricultural products in the study areas.

In this study, one soil sample was collected per km^2^ in the surface layer (0–20 cm), totaling 2023 samples. Agricultural product samples were obtained from local markets, and drinking water samples were collected from wells throughout the entire study region. The sampling sites of 2023 soil samples, 274 drinking water samples, and 85 agricultural product samples (including canola seeds, corn, tea, and rice) are depicted in Figure 2.

The soil samples were air-dried and finely ground into a powder using a 100-mesh nylon sieve. They were then stored in plastic bags at 25 °C for later chemical analysis. Similarly, the agricultural product samples were rinsed and oven-dried at 60 °C until they reached a constant weight. These samples were also ground into powders and stored in plastic bags at 25 °C.

For concentrations of total Selenium analysis, soil and agricultural product samples were subjected to acid digestion using HNO_3_-HClO_4_ (3:2, *v*/*v*) and HNO_3_-HClO_4_ (4:1, *v*/*v*), respectively. In the case of drinking water samples, their pH values were adjusted down to 2 by adding high-purity HNO_3_. The total selenium content in soil, agricultural products, and drinking water was determined using an inductively coupled plasma mass spectrometry (ICP-MS) instrument (NexION 2000, PerkinElmer, Waltham, MA, USA). Furthermore, the total fluoride content in soil was determined using the high-temperature pyrohydrolysis fluoride ion electrode method (SevenExcellence, Mettler Toledo, Greifensee, Switzerland), following the standard method (HJ 873-2017) [13]. The total fluoride content in drinking water was determined based on the standard method (GB/T 7484-1987) [14].

### 2.3. Statistical Analysis

The research utilized ArcGIS software version 10.7 (Zondy Cyber Group Co., Ltd., Wuhan, China) to map sampling points, analyze the spatial distribution of DFI, and perform spatial statistical analysis. SPSS version 19.0 was employed for statistical analysis, such as calculating averages, standard deviations, and other relevant metrics. Statistical significance was determined at the level of *p* < 0.05. The generalized additive model (GAM) [15] was applied to identify non-linear relationships between soil selenium, fluoride, selenium/fluoride content, and DFI. GAM was fitted in R (v4.4.3) using mgcv. Thin-plate regression splines were used; smoothing parameters were selected by REML. Model diagnostics were examined and effects are shown with 95% confidence intervals. Selenium and fluoride concentrations in soil, agricultural products, and drinking water samples were reported as means ± standard deviations and medians. Spearman’s correlation analysis was used to assess the correlations between selenium content, fluoride content, selenium/fluoride ratio in soil and drinking water, and DFI. Moran’s index (Moran’s I) was utilized to examine the overall spatial correlation. Global autocorrelation analysis was employed to describe the spatial distribution of attribute values across the entire study area, comparing each value to the mean value to derive the correlation. Moran’s I index was calculated using the following formula [16].

$$I=n\sum_{i=1}^{n}\sum_{j=1}^{n}\omega_{ij}(x_{i}-\bar{x})(x_{j}-\bar{x})/(\sum_{i=1}^{n}\sum_{j=1}^{n}\omega_{ij})\sum_{i=1}^{n}{\left(x_{i}-\bar{x}\right)}^{2}$$

*n* is the number of regions; *x_i_* and *x_j_* are the attribute values at area *i* and *j*; $\bar{x}$ is the mean value of the attribute in the study region; and *w_ij_* is the element of the spatial weight matrix between area *i* and *j*, representing the spatial relationship between them. The range of Moran’s I statistics is from −1 to 1. A positive value of Moran’s I indicates a positive spatial correlation, while a negative value indicates a negative spatial correlation. The absolute value of Moran’s I closer to 1 implies a stronger spatial correlation. When Moran’s I equals 0, it signifies no spatial correlation between the values analyzed [17].

### 2.4. Getis–Ord General G

Getis–Ord statistics is a distance-based tool to measure the proportion of a variable within a given radius of a point to that in the whole study region. The statistics for location *i* are defined as

$$G_{i}=\frac{\sum_{j=1}^{n}w_{ij}(d)x_{j}}{\sum_{i=1}^{n}x_{i}}$$

*x_j_* is the observed value at point *j*; *w_ij_(d)* is the *ij* element of a binary W matrix (*w_ij_* = 1 if within distance d, *w_ij_* = 0 if elsewhere); and n is the observational number. The statistical mean and variance can be used to obtain a standard statistical value. When the value is significantly greater than the cut-off value, there is a positive or negative spatial association. Positive values represent spatial agglomeration. The higher the Z-score, the higher the possibility of clustering. A Z-score close to 0 means there is no obvious cluster [3,17].

## 3. Results

### 3.1. Spatial Geochemical Distribution Pattern of Environmental Selenium and Fluoride

#### 3.1.1. Selenium and Fluoride in Soil and Drinking Water

Soil selenium content varied from 0.06 to 14.30 mg·kg^−1^, with 0.78 ± 0.66 mg·kg^−1^ as the mean value (Table 1). Notably, over half of the drinking water samples fall below the instrument’s detection limit, with the highest recorded value at 2.68 × 10^−2^ mg·L^−1^. The distribution of soil selenium content is uneven and site-specific, deviating significantly from the national background value of 0.239 mg·kg^−1^. Tan’s classification designates the study area as a selenium-enriched region, with 77.9% of soil selenium content above 0.45 mg·kg^−1^ [18]. In 274 drinking water samples, selenium content varied from nd to 2.68 × 10^−2^ mg·L^−1^, with 70 × 10^−3^ mg·L^−1^ as the mean.

The soil samples show significant variability in fluoride content, ranging from 84.00 to 6.93 × 103 mg·kg^−1^, with an average of 1.11 × 10^3^ ± 7.15 × 10^2^ mg·kg^−1^ and a median of 9.11 × 10^2^ mg·kg^−1^. Drinking water samples exhibit lower fluoride levels, with the total fluoride content ranging from 1.58 × 10^−2^ to 2.53 mg·L^−1^, with an average of 0.21 ± 0.26 mg·L^−1^ and a median of 0.15 mg·L^−1^.

#### 3.1.2. Agricultural Products and Selenium

The average selenium content in various agricultural products follows an ascending order (Table 2): canola seeds (65.8 µg·kg^−1^), rice (60.3 µg·kg^−1^), tea (50.1 µg·kg^−1^), and corn (28.3 µg·kg^−1^). Variations in selenium absorption and accumulation among different plants contribute to this hierarchy. Despite the enriched soil selenium content, the total selenium content in various foods generally does not exceed levels found in other regions of China.

#### 3.1.3. Spatial Distribution of Selenium, Fluoride, and Selenium/Fluoride in Soil and Drinking Water

**Figure 3:**
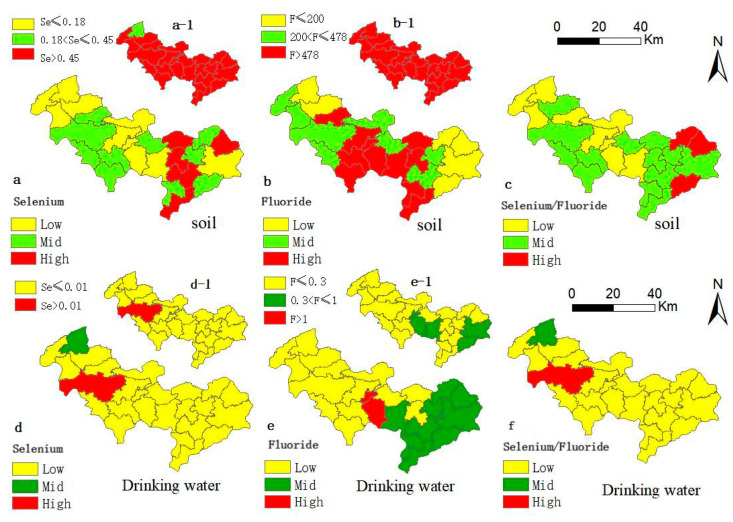
Color-graded geochemical distribution maps of selenium content (**a**), fluoride content (**b**), and selenium/fluoride (**c**) in soil, and selenium (**d**), fluoride (**e**), and selenium/fluoride ratio (**f**) in drinking water (unit: mg·kg^−1^ for soil, mg·L^−1^ for drinking water). Note: (**a**–**f**)are color-graded geochemical distribution maps dividing the selenium, fluoride, or selenium/fluoride content in all soil samples into Low, Mid, and High categories, while (**a-1**,**b-1**,**d-1**,**e-1**) are color-graded geochemical distribution maps based on the established criteria for selenium and fluoride in soil and drinking water as reported in the literature [4,18,24,25].

The geochemical distributions of selenium content, fluoride content, and selenium/fluoride ratio in soil and drinking water are shown in Figure 3. The significantly elevated levels of selenium is observed in most towns within the study area, with the exception of one town in the northwest corner having the lowest soil selenium content (<0.18 mg·kg^−1^) (Figure 3(a-1)). Intriguingly, the severity of dental fluorosis appears more pronounced in this region, suggesting a potential link between lower soil selenium levels and increased dental fluorosis severity. Furthermore, soil fluoride contents in all towns surpass soil background values (>478 mg·kg^−1^) [23,24] (Figure 3(b-1)). The majority of the study area could be classified as enriched in soil selenium and high in fluoride content. Mid-eastern areas exhibit high soil selenium content, while middle areas display a concentration of high soil fluoride content. Geochemical distribution maps for selenium and fluoride in drinking water reveal low selenium content and selenium/fluoride ratio, with elevated fluoride levels mainly in the eastern areas. Interestingly, most towns, except for one with the lowest soil selenium content, exhibit selenium in significantly high states.

#### 3.1.4. Geochemical Anomalies and Limited Transfer

The geochemical distribution of fluoride and selenium in soil reveals anomalies suggestive of significantly high states. Although soil selenium and fluoride were geochemically elevated, transfer to water and crops was limited, implying alternative exposure pathways. The potential source of significant fluoride release, involving clay soil in coal combustion, raises concerns about food contamination. Importantly, there is no conclusive evidence suggesting a similar exposure pathway for selenium as for fluoride, as discussed in the relevant literature. These findings underscore the complexity of environmental processes and the need for a comprehensive understanding of element mobility.

### 3.2. Correlations and Spatial Association of Selenium or Fluoride with DFI

#### 3.2.1. Correlations with DFI

The study reveals correlations between environmental selenium and fluoride levels with DFI. The correlation between soil selenium content and DFI was weakly negative (R = −0.233, *p* < 0.01). Soil fluoride content was statistically association with DFI (R = 0.086, *p* < 0.01), the correlation strength was extremely weak. Additionally, there was also extremely weak correlation between soil selenium/fluoride and DFI (R = −0.085, *p* < 0.01).

Spatial statistics can be used to examine spatial distribution patterns and identify local characteristics and developmental imbalances. Spatial correlations were explored, following the first law of geography [26]. Spatial autocorrelation was analyzed using Moran’s I statistics. Moran’s I statistics for selenium, fluoride, selenium/fluoride ratio in soil, and DFI ranged from 0.41 to 0.48. Additionally, the absolute values of the Z-scores were greater than 2.58, indicating statistical significance (*p* < 0.05), suggesting statistically significant spatial aggregation emphasizing non-random distribution patterns.

#### 3.2.2. Association with DFI

**Figure 4:**
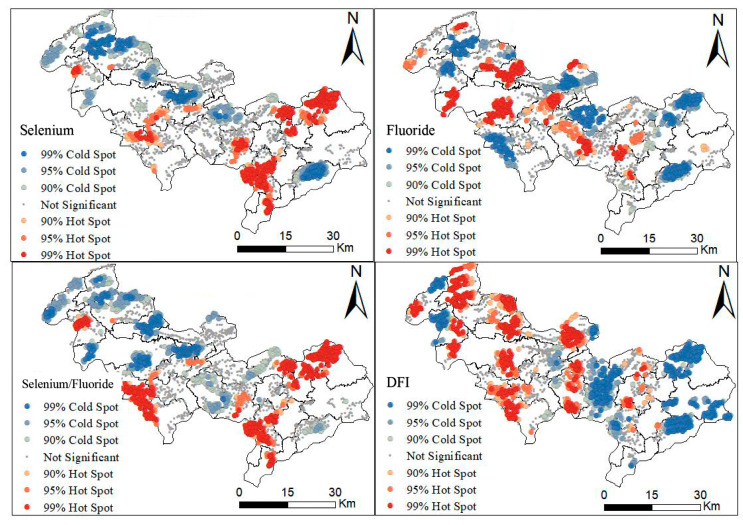
Distribution of cold-/hot-spots for soil selenium, fluoride, selenium/fluoride, and DFI in the study area.

In order to further investigate the effect of soil selenium, fluoride, and selenium/fluoride on DFI, Getis-Ord General G analysis was employed to identify clustering patterns in high-value regions [4]. Areas with G_i_ values of < 0.01 reveal spatial distribution anomalies. The areas with G_i_ values of <0.01 are depicted in Figure 4. The General G observation index exceeded the General G expectation index and Z was >+2.58, suggesting clustering in high-value regions. The hot spots of soil selenium were mainly distributed in the mid-east zone, while the cold spots were mainly present in the northern and central zones. The hot spot area of soil fluoride was located in the mid-eastern zone, while the cold spot area was concentrated in the northwestern zone. The hot spot area of DFI was found in the mid-western zone, while the cold spot area was mainly distributed in the mid-eastern zone. The spatial distribution of soil selenium/fluoride coincided with the hot/cold spots of soil selenium. This suggested a reverse relationship between soil selenium or selenium/fluoride and DFI.

### 3.3. The Generalized Additive Model (GAM) of Soil Selenium Content, Fluoride Content and Selenium/Fluoride with the Dean’s Dental Fluorosis Index

**Figure 5:**
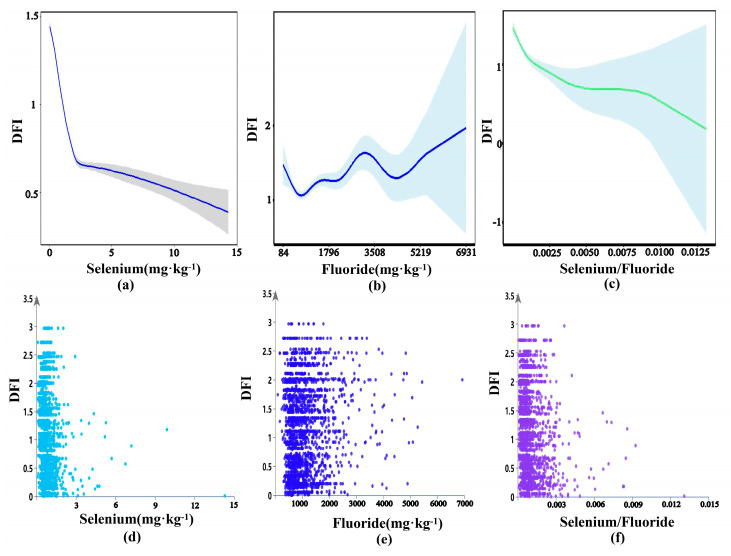
The non-linear effect and scatter-plot diagrams of DFI versus soil selenium content (**a**,**d**), fluoride content (**b**,**e**), and selenium/fluoride (**c**,**f**).

In this study, a negative correlation was observed between soil selenium and the DFI. However, it is important to note that the analysis only involved simple intergroup comparisons and did not account for continuous variation in the effects of selenium content on DFI. The generalized additive model (GAM) can be applied to reveal the relationships between environmental factors. Scatter-plots and GAM analysis were conducted to analyze DFI associations with soil selenium content (Figure 5a,d), soil fluoride content (Figure 5b,e), and the soil selenium/fluoride ratio (Figure 5c,f).

The results of the scatter-plot analyses indicated that there was no linear relationship between DFI and soil selenium content, fluoride content, or the selenium/fluoride ratio. The relationship between soil fluoride content and DFI showed a “W” pattern (Figure 5b). Initially, as fluoride content increased from 84.00 to 8.00 × 10^2^ mg·kg^−1^, DFI decreased. A significant peak in DFI occurred between 8.00 × 10^2^ and 4.0 × 10^3^ mg·kg^−1^ of fluoride content. Subsequently, DFI was observed as soil fluoride content increased above 4.00 × 10^3^ mg·kg^−1^. The fitting of the data using generalized additive parameters yielded a smoothing parameter of 0.6, with 26 degrees of freedom and 2023 observations, which were determined to be statistically significant at α = 0.05. The relationship between soil selenium content and DFI is illustrated in Figure 5a. Initially, when soil selenium content was below 2.30 mg·kg^−1^, DFI decreased steeply. However, as soil selenium content changed, DFI decreased gradually. The relationship between soil selenium/fluoride ratios and DFI also exhibited a similar pattern (Figure 5c) to that observed with soil selenium content.

## 4. Discussion

Guizhou Province in Southwest China, is a typical carbonate area and has a high fluoride background. The surface instability of exposed carbonate bedrock can lead to the release of fluorine into the water, soil, and air and thereby, into the biogeochemical cycle. Soil fluoride levels are higher than in China (478 mg·kg^−1^) and the world (200 mg·kg^−1^) [27]. High levels of fluoride in the environment might pose a health risk to humans [24]. The average fluoride content in soil (1100 mg·kg^−1^) in the study area is markedly higher than in other locations globally, raising environmental concerns. The study area has a typical subtropics Karst landform, which is controlled by the superposition of weathering and erosion of carbonate rocks and the coal-bearing strata [28]. In the Indo-Gangetic plains, the average fluoride level of topsoil was 515 mg·kg^−1^ [29]. In Central Pomerania, Poland, the average was 3.79 mg·kg^−1^ [30]. In the Qingshui River plains, Ningxia Province, China, the average level of fluoride of surface soil was about 520 mg·kg^−1^ [31]. In Dali County, Northwest China, soil fluoride contents ranged from 1.18 mg·kg^−1^ to 13.70 mg·kg^−1^ [22]. In China, the average fluoride content of topsoil is 478 mg·kg^−1^, and the critical value of fluoride epidemic value was 800 mg·kg^−1^ [32,33,34,35].

Soil selenium distribution is extremely uneven and site-specific. The range of soil selenium content in China was 0.005–79.08 mg·kg^−1^ [23,36]. In the study area, the average of soil selenium is significantly higher than the national background value of 0.239 mg·kg^−1^ [25]. Tan proposed that soil total selenium content was classified as five levels (mg·kg^−1^): deficient (<0.18); moderate (0.18–0.45), enriched (0.45–2.0), high (2.0–3.0), and toxic (>3.0) [18]. Based on Tan’s classification, 77.9% of soil selenium content were above 0.45 mg·kg^−1^ indicating that the study area was actually a selenium-enriched region.

This study also showed that the average water fluoride content in the study area was 0.21 mg·L^−1^, which was far below China’s standards for drinking water quality (1 mg·L^−1^) (GB 5749-2022) [37]. It suggested that the amount of fluoride ingested by local residents through drinking water was unlikely to be the main route of fluoride exposure in this zone. Selenium content from drinking water with an average of 4.7 × 10^−3^ mg·L^−1^ was the range of soil selenium content in China and was significantly lower compared with European and Chinese surface water selenium standards (0.01 mg·L^−1^) (GB 5749-2022) [37,38]. It was also lower than the WHO standard and American standard (0.050 mg·L^−1^). Therefore, although the soil selenium was enriched, selenium content in drinking water was relatively low.

Previous studies have shown a close relationship between fluorosis and abnormal levels of elements, such as fluoride from the environment [23,39]. Earlier research also have reported that fluoride levels in soil, food, or water, as well as fluoride exposure are associated with the severity of fluorosis [40]. For example, Hao et al. investigated the relationship between soil chemical elements and blood samples from individuals suffering from coal-burning fluorosis and found a positive correlation between soil fluoride content and fluorosis. Although soil fluoride content and DFI showed a very weak but statistically significant association, there was a weakly negative correlation between soil selenium content and DFI. Furthermore, spatial autocorrelation analysis revealed that there was statistically significant spatial aggregation emphasizing non-random distribution patterns for soil fluoride, selenium, and selenium/fluoride (*p* < 0.05). Based on the Getis-Ord General G analysis, hot/cold spots suggested there was a reverse relationship between soil selenium or selenium/fluoride and DFI.

It has been confirmed that the main source of coal-burning pollution-induced fluorosis in most areas is clay. However, the dosage of fluoride intake and the severity of fluorosis are also closely related to people’s habits [41]. Therefore, some observations have found that there was no significant correlation between the prevalence of coal-burning pollution-induced fluorosis and the geochemical background of clay fluoride under large-scale conditions [5]. However, in the selected study area, which is predominantly within the same county, people’s habits are very similar. Therefore, there is a correlation (*p* < 0.05) between DFI and the geochemical background of clay selenium, indicating selenium in clay was one of the leading factors of alleviating dental fluorosis.

Selenium had an antagonistic effect on the development of dental fluorosis [42]. Low selenium levels in the environment and low selenium nutrition in the population may aggravate the tea-drinking fluorosis in Tibet [43]. There were negative correlations between children’s dental fluorosis prevalence and surface soil selenium content [44]. Selenium is a crucial component of numerous enzymes and proteins and has been demonstrated to reduce fluorosis symptoms and urinary fluoride excretion in rats exposed to high fluoride concentrations [45]. Selenium may alleviate the severity of dental fluorosis mainly based on the fluoride-induced oxidative stress injury. It was reported that long-term consumption of antioxidants and plant products could reduce the severity of the clinical symptoms of fluorosis or avoid fluorosis [7,46]. Selenium intervention can reduce the apoptosis of renal cells induced by fluorosis in rats [8].Selenium can mitigate fluorosis by improving estrogen levels [47].

## 5. Conclusions

The investigation into the spatial correlation of environmental fluoride and selenium in coal-burning fluorosis areas of Southwest China provides comprehensive insights into their implications for dental fluorosis. In exploring the spatial geochemical distribution patterns, the study reveals significant variability in soil selenium content, emphasizing the complexity of exposure pathways. Despite the classification of the study area as selenium-enriched, agricultural products display nuanced absorption, reflecting varied accumulation among different plants. In contrast, soil fluoride content exceeds global averages, raising environmental concerns. Correlations with dental fluorosis reveal a distinctive “W” pattern, highlighting the intricate impact of fluoride content on DFI. Significantly high patterns in selenium, coupled with spatial clustering and a reverse relationship in identified hot/cold spots, underscore the influence of local geological factors on dental health outcomes. The application of the GAM unravels complex, non-linear relationships, emphasizing optimal ranges mitigating fluorosis risk. These findings, situated against the background of coal-burning fluorosis in Southwest China, carry implications for region-specific public health interventions. Recognizing the interplay of environmental factors in dental health outcomes, this study prompts further research to unravel the complexities of these associations and inform targeted health policies for coal-burning fluorosis regions in Southwest China. Despite limitations, including the lack of individual intake assessment and longitudinal follow-up to analyze water samples, this study contributes to our understanding of the dynamic relationships between geochemical factors and dental fluorosis, urging continued efforts to address the multifaceted challenges posed by coal-burning fluorosis areas.

## Figures and Tables

**Table 1:** Descriptive statistics of selenium and fluoride concentration in different samples. (Unit: mg·kg^−1^ in soil, mg·L^−1^ in drinking water).

Type	Selenium					Fluoride					
	n	Min	Max	Means	SD	Median	Min	Max	Means	SD	Median
Soil	2023	0.06	14.30	0.78	0.66	0.64	84.00	6.93 × 10^3^	1.11 × 10^3^	7.15 × 10^2^	9.11 × 10^2^
Drinking water	274	nd	2.68 × 10^−2^	4.70 × 10^−3^	2.10 × 10^−2^	nd	1.58 × 10^−2^	2.53	0.21	0.26	0.15
Total	2297	0.06	14.30				1.58 × 10^−2^	6.93 × 10^3^			

nd = not detected (below LOD).

**Table 2:** Selenium contents of different agricultural products in the study area (Unit: µg·kg^−1^).

Type	n	Min	Max	Means	SD	Reference Ranges
Tea	16	19.0	145.1	50.1	30.3	440 [19]
Rice	36	9.0	125.6	60.3	21.5	90 [20]
Canola seeds	17	36.9	122.0	65.8	21.2	187.5 [21]
Corn	16	12.0	50.0	28.3	13.1	4040–7720 [22]

## Data Availability

The raw data supporting the conclusions of this article will be made available by the authors on request.
